# Supplementary material for: Identifying fishing grounds from vessel tracks: model-based inference for small scale fisheries
Source: R Soc Open Sci. 2019 Oct 2;6(10):191161. doi: 10.1098/rsos.191161 (PMC6837222; doi:10.1098/rsos.191161)
Supplement: Supplementary figures [file rsos191161supp1.pdf]

Supplementary material S1

The figures presented here are a supplement to "Identifying fishing grounds from vessel tracks: model-based inference for small scale fisheries" by Tania Mendo, Sophie Smout, Theoni Photopoulou and Mark James (2019) Royal Society Open Science.

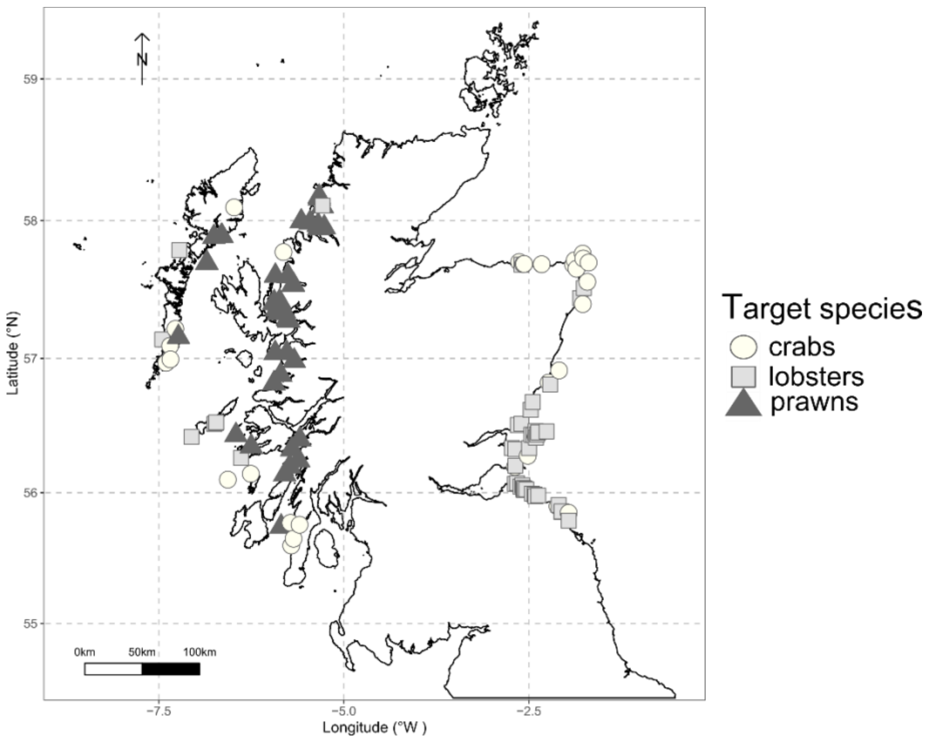

Fig. S1.1 – Map showing locations of fishing trips with on-board observers. White circles show trips targeting crabs (brown and velvets), grey squares where the main species was lobster, and dark grey triangles where the main target species was prawns.

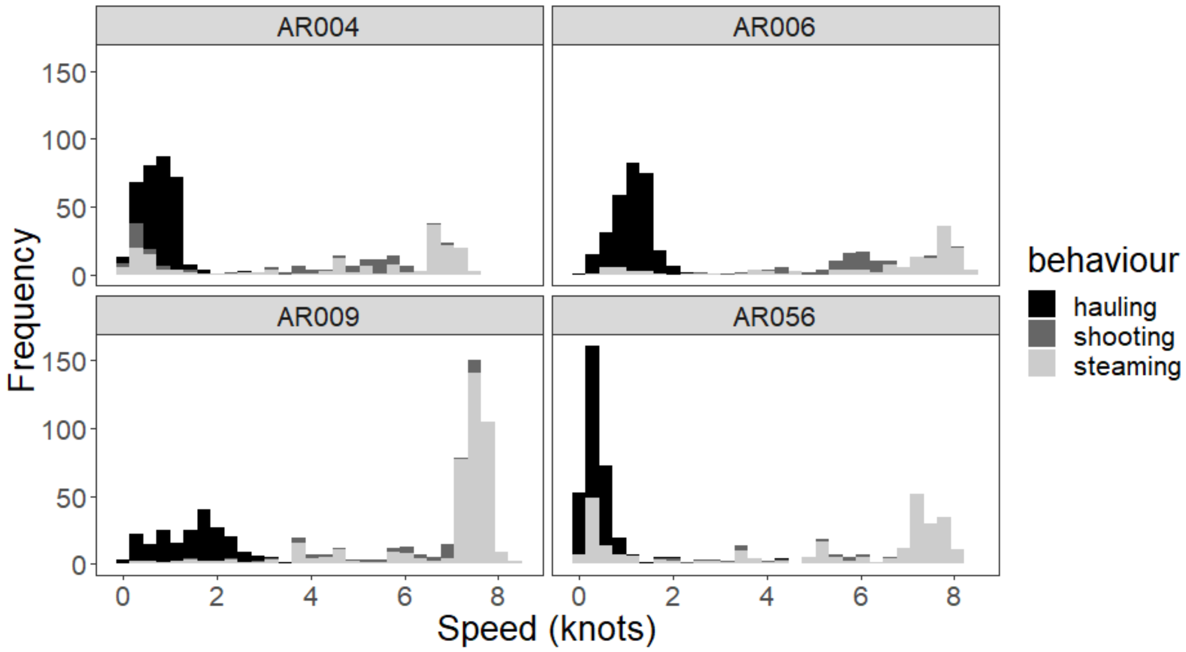

Fig. S1.2. Frequency distribution of vessel speed (knots) in each behaviour (hauling, deploying or shooting, steaming) showing how speeds during deployment can overlap with speeds during steaming.

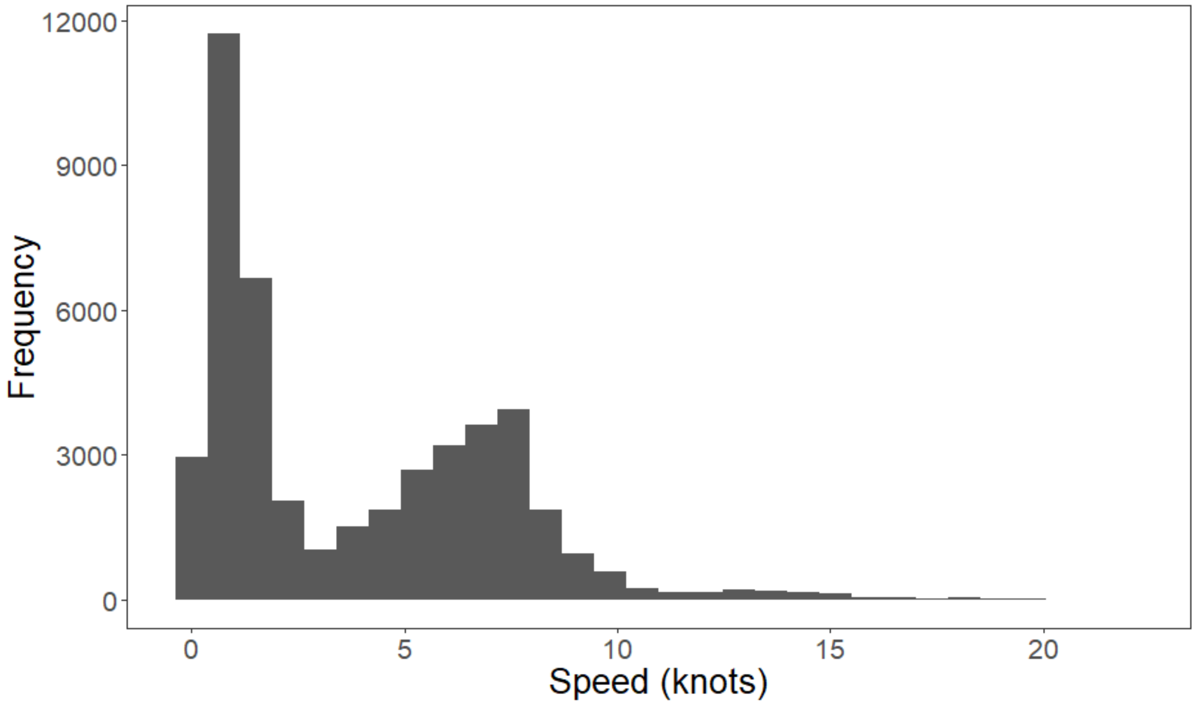

Fig. S1.3. Multimodal distribution of speed used for estimation of mean and standard deviation for each underlying behaviour (hauling mean=2, sd=2, deploying mean=6, sd=2, steaming=14, sd=2)

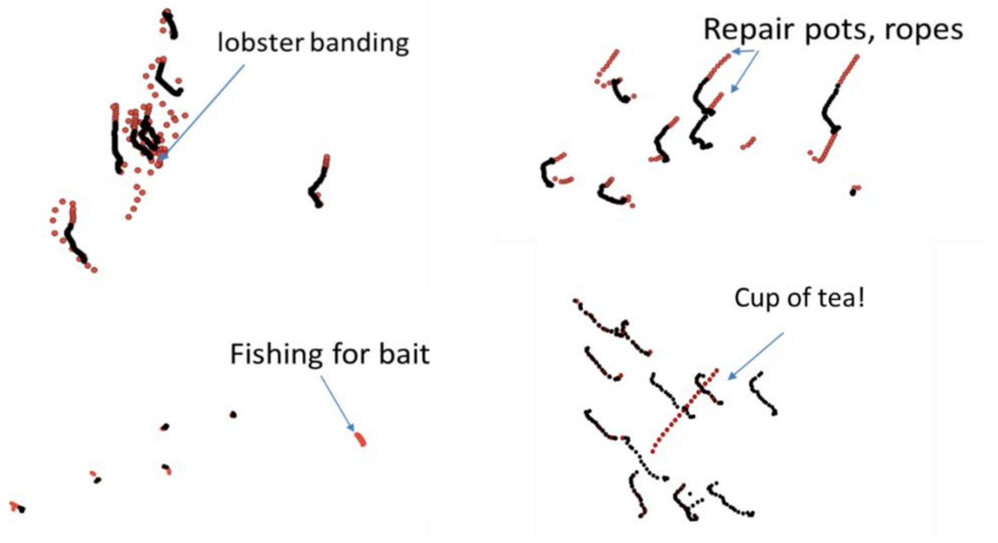

Fig. S1.4. Main reasons for false positives (red circles) in identifying hauling events (black circles): lobster banding after a haul, repairing ropes or pots, fishing for bait or stopping for lunch or to drink a cup of tea.

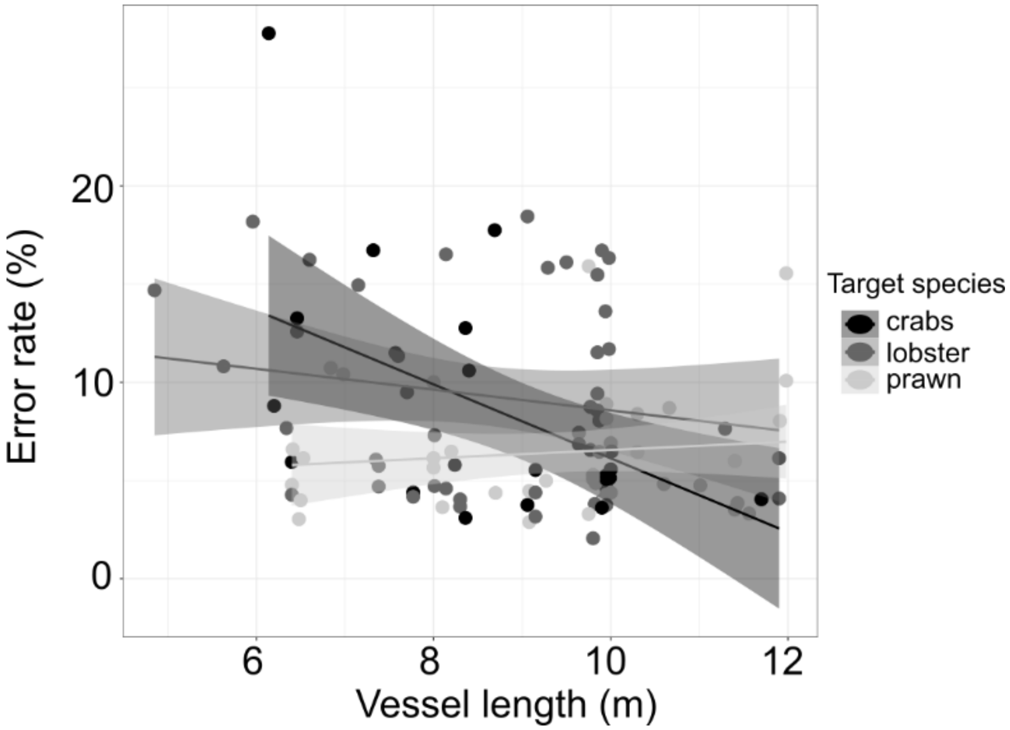

Fig. S5. Relationship between vessel length (m) and error rate (%) for each trip targeting either crabs, lobsters, or prawns.
