# Supplementary material for: Identifying fishing grounds from vessel tracks: model-based inference for small scale fisheries
Source: R Soc Open Sci. 2019 Oct 2;6(10):191161. doi: 10.1098/rsos.191161 (PMC6837222; doi:10.1098/rsos.191161)
Supplement: R code for carrying out the analysis [file rsos191161supp2.pdf]

Supplementary Material S2

Identifying fishing grounds from vessel tracks: model-based inference for small scale fisheries

22 August 2019

The code presented here is a supplement to "Identifying fishing grounds from vessel tracks: model-based inference for small scale fisheries" by Tania Mendo, Sophie Smout, Theoni Photopoulou and Mark James (2019) Royal Society Open Science.

In this document we compare five different methods for identifying hauling versus non-hauling activities in small scale fishing vessels from their movement trajectories. These are described in detail in the main text of the paper.

We provide a sample dataset of movement data collected every 60sec from 5 trips by 5 different small scale fishing vessels using creels. The data can be found here, DOI: https://doi.org/10.5061/dryad.k80bp46

First, read in and process the data for analysis

We use the `moveHMM` package to compute the distances and turning angles between consecutive location records. Remove records with missing step length (we assume they are missing at random), and convert the step length values from meters to knots (nautical miles per hour).

```
# load moveHMM package
library(moveHMM)

# read in a .txt file containing the data
sdata <- read.table("example_data.txt", header=TRUE, sep=",")
table(sdata$ID)

# use the prepData function to create step lengths
prepdat <- moveHMM::prepData(sdata, type = "UTM", coordNames = c("x", "y"))
# check if there are any missing step lengths
# there will be at least five (=the number of unique IDs) because the first step
# length of each trip will be NA
table(is.na(prepdat$step))

# remove missing step lengths
prepdat <- prepdat[!is.na(prepdat$step),]

# convert step length from meters to knots (nautical miles per hour)
prepdat$speed <- prepdat$step/60*1.9438
# look at the histogram of speeds
hist(prepdat$speed, xlab="Speed in knots")
```

1. "Overall" speed threshold using a Gaussian mixture model

This method uses a Gaussian mixture model to group the speed of all fishing vessels (i.e., overall) in to two categories: hauling or not hauling. We know there to be three true, functional states from observation records: hauling gear, deploying or shooting gear, and steaming between fishing locations. Although we fit a model with these three states, we are only interested in the binary labelling of hauling vs not hauling, so we group the two non-hauling states together. We fit this model using the `normalmixEM` function in the `mixtools` `R` package.

```
# load mixtools package
library(mixtools)

# use the histogram of speeds from all trips (generated above in the data preparation
# chunk) to get estimates of starting values.
# we chose speeds of 1, 4 and 8 nautical miles for hauling gear, deploying gear and
# steaming, and we chose standard deviations of 1 for all states.
# we then fit a Gaussian mixture model
EM_all <- normalmixEM(prepdat$speed, mu = c(1,4,8), sigma = c(1,1,1))

# plot the estimates to if they make sense
plot(EM_all, density=TRUE,loglik=FALSE)

# assign a maximum value for the speed during hauling behaviour.
# here we use the estimated hauliung mean from EM_all and add 1.96 times the
# standard deviation for the hauling distribution, identified by the
# mixture model using an EM algorithm, above.
speed_threshold_EM <- EM_all$mu[1]+1.96*EM_all$sigma[1]

# assign the overall behaviour as hauling or not hauling according to the
# speed threshold defined above.
prepdat$overall_EM_beh <- ifelse(prepdat$speed < speed_threshold_EM,
                                  "hauling", "not_hauling")
```

2. Trip-based Gaussian mixture model

Here we use a method similar to 1. above, but instead of fitting a single model to the data from all trips, we fit a model to each trip and combine the results.

```
# load dplyr package
library(dplyr)

# split the data into trips (total 5 trips)
in.list2 <- split(prepdat, prepdat$ID)
out.list2 <- list()
N <- length(in.list2)

# loop through each trip, fitting a Gaussian mixture model as in 1.
for (i in 1:N){

trip_EM <- normalmixEM(in.list2[[i]]$speed, mu = c(1,4,8), sigma = c(1,1,1))
ks <- (unlist(c(trip_EM$lambda, trip_EM$mu, trip_EM$sigma, trip_EM$loglik)))
ks <- as.data.frame(t(ks))
out.list2[[i]] <- ks

if (sum(ks) != 0 ) {

  print(paste("i=",i))
  plot(trip_EM, density=TRUE,loglik=FALSE)

  } else {

      (paste("i=",unique(in.list2[[i]]$ID), "is 0. Not processed")) # Print out progress
```

```
    next
  }
}

# turn the out.list2 object back into a dataframe, this includes the estimated
# parameters for all trips
trip_EM_par <- bind_rows(out.list2)
colnames(trip_EM_par) <- c("lambda1", "lambda2", "lambda3","mu1", "mu2", "mu3","sd1",
                           "sd2", "sd3","loglik")

# add trip ID to trip_EM parameter dataframe
trip_EM_par$ID <- unique(prepdat$ID)

# merge the dataframe holding the parameter estimates and the data
prepdat <- merge(prepdat, trip_EM_par[,c("ID","mu1","sd1")], by=c("ID"))

# create the hauling speed upper limit and assign states to locations using this threshold
speed_threshold_trip_EM <- prepdat$mu1 + 1.96 * prepdat$sd1
prepdat$trip_EM_beh <- ifelse(prepdat$speed < speed_threshold_trip_EM, "hauling",
                                                                        "not_hauling")

# remove unnecesary columns
names(prepdat)
prepdat <- prepdat %>%
  select(-c("mu1","sd1"))
```

3. Binary Clustering for behavioural annotation using Gaussian mixture models on a trip-by-trip basis

The third approach we use is also a Gaussian mixture model but

```
# load EMbC package
library(EMbC)

prepdat$date <- as.POSIXct((prepdat$date), format = "%Y-%m-%d %H:%M:%S")

# transform UTM coordinates to WGS84 (lon, lat)
xy <- data.frame(X = prepdat$x, Y = prepdat$y)
library(sp)
 coordinates(xy) <- c("X", "Y")
 proj4string(xy) <- CRS('+proj=utm +zone=30 +datum=WGS84 +units=m
                        +no_defs +ellps=WGS84 +towgs84=0,0,0')  # for example
 coor <- as.data.frame(spTransform(xy, CRS("+proj=longlat +datum=WGS84")))
prepdat$lon <- coor$X
prepdat$lat <- coor$Y

# choose relevant columns for analysis: time stamp, x and y
names(prepdat)
prepdat3 <- prepdat %>%
  select("ID","date","lon","lat")

EMbc_value <- data.frame()
out.list3 <- split(prepdat3, prepdat3$ID)
```

```r
# loop through the trips in out.list2, by name
for (ii in names(out.list3)){
    iidf <- as.data.frame(out.list3[[ii]][,2:4])
    # carry out speed/turn bivariate binary clustering for each trip, using stbc()
    EMbc <- stbc(iidf, info=-1)
    # take the numeric vector of clustering labels (A) and turn them into a data frame
    EMbcA <- as.data.frame(EMbc@A)
    # attach the trip ID to the data frame
    EMbcA$trip_ID <- ii
# plot clustering visually
sctr(EMbc)
# plot track with labels
view(EMbc)
# print trip label for sense-check
print(ii)
# attach trip-specific output to main outout data frame
EMbc_value <- rbind(EMbc_value, EMbcA)
}

dev.off()
graphics.off()

# hauling events are associated with low speeds, low turning angles
EMbc_value$Embc_simple <- ifelse(EMbc_value$`EMbc@A`==1 | EMbc_value$`EMbc@A`==2,
                                  "hauling", "not_hauling")

# attach results to the main data set and check it looks ok
prepdat$trip_EMbc_beh <- EMbc_value$Embc_simple
head(prepdat)
```

4. Hidden Markov model with speed only

The next two approaches employ hidden Markov models implemented in the R package moveHMM. We first fit a model to speed only. We use parameters from trip-based Gaussian mixture model fitted in 2. above, as initial parameters for gamma distributions of step length (distance travelled between regular observations in time).

```r
# load the moveHMM and dplyr package
library(moveHMM)
library(dplyr)

# split the data into trip (total 5 trips)
in.list4 <- split(prepdat, prepdat$ID)
out.list4 <- list()
N <- length(in.list4)

# loop through trips to find starting values using a Gaussian mixture model
# (as in 2, above) to do so
for (i in 1:N){

trip_EM <- normalmixEM(in.list4[[i]]$step, mu = c(20,150,300), sigma =c(20,20,20))
ks <-(unlist(c(trip_EM$lambda, trip_EM$mu, trip_EM$sigma, trip_EM$loglik)))
ks <- as.data.frame(t(ks))
out.list4[[i]] <- ks
```

```r
if (sum(ks) != 0 ) {

  print(paste("i=",i))

  } else {

      (paste("i=",unique(trip_EM[[i]]$ID), "is 0. Not processed")) # Print out progress
      next
  }
}

# combine the out.list4 object back into a dataframe
trip_EM_par_step <- bind_rows(out.list4)
head(trip_EM_par_step)
colnames(trip_EM_par_step) <- c("lambda1", "lambda2", "lambda3",
                                "mu1", "mu2", "mu3",
                                "sd1", "sd2", "sd3",
                                "loglik")

# add trip ID to trip_EM_par_step dataframe
trip_EM_par_step$ID <- unique(prepdat$ID)

# now fit HMM using the trip-specific GMM estimates for speed as starting values

library(purrr)

# I have to create a moveData object again - but duplicate remove step and angle
in.list4hmm <- split(prepdat, prepdat$ID)
out.list4hmm <- map(in.list4hmm, function(x) select(x, -c(step, angle, id))) %>%
  map(., function(x) prepData(x, type = "UTM", coordNames = c("x", "y")))
head(out.list4hmm[[1]])

N <- length(out.list4hmm)

for (i in 1:N){
# to initiate numerical maximization (optimization of the likelihood) starting values
# for the model parameters need to be specified.

  mu0 <- c(trip_EM_par_step$mu1[i],
              trip_EM_par_step$mu2[i],
              trip_EM_par_step$mu3[i])
  sigma0 <- c(trip_EM_par_step$sd1[i],
             (trip_EM_par_step$sd2[i]-trip_EM_par_step$sd2[i]/4),
             (trip_EM_par_step$sd3[i]+2))

  # we specify a smaller SD for speed during deployment behaviour than during steaming

stepPar0 <- c(mu0,sigma0)
stepDist <- "gamma"

# fit an HMM with 3 states
m <- fitHMM(data=out.list4hmm[[i]], nbStates=3, stepPar0=stepPar0,
            verbose=0, stepDist=stepDist, angleDist='none')
```

```
# print(m)
ks <- as.data.frame(unlist(m$mle))
vit <- viterbi(m)

if (sum(ks) != 0 ) {
  write.table(ks, paste0(trip_EM_par_step$ID[i],".txt"))
  write.table(vit, paste0('viterbi_speed',trip_EM_par_step$ID[i],".txt"))
  print(paste("i=",i))
  plot(m,compact=TRUE,ask=FALSE)#the compact bit puts all vessels in one graph

# compute the pseudo-residuals
#pr <- pseudoRes(m)
# time series, qq-plots, and ACF of the pseudo-residuals
#plotPR(m)

  } else {

      print(paste("i=",i, "is 0. Not processed")) # Print out progress
      next
    }
}

dev.off()
graphics.off()

list.filenames <- list.files(path = ".", full.names = FALSE, pattern="^viterbi_speed")
datalist <- map(list.filenames, function(x) read.table(x, header=T, sep=""))
names(datalist) <- list.filenames
trial <- do.call(rbind.data.frame, datalist)
prepdat$viterbi_speed <- trial$x #2221 obs

prepdat$trip_HMM_speed_beh <- ifelse(prepdat$viterbi_speed==1, "hauling", "not_hauling")
```

5. Hidden Markov Model with speed and turning angle

In the second HMM approach we fit a model to speed and turning angle. We use parameters from trip-based Gaussian mixture model fitted in 2. above, as initial parameters for the mean of the gamma distributions of step length (distance travelled between regular observations in time). We provided a smaller standard deviation for hauling and deploying gear than for steaming. For the turning angle we chose starting values to reflect that we expected hauling to have a larger turning angle than steaming.

```
# load the moveHMM package
library(moveHMM)

# split the data into trip (total 5 trips)
in.list5hmm <- split(prepdat, prepdat$ID)
out.list5hmm <- map(in.list5hmm, function(x) select(x, -c(step, angle, id))) %>%
  map(., function(x) prepData(x, type = "UTM", coordNames = c("x", "y")))
N <- length(in.list5hmm)

for (i in 1:N){

  mu0 <- c(trip_EM_par_step$mu1[i],
```

```
           trip_EM_par_step$mu2[i],
           trip_EM_par_step$mu3[i])
  sigma0 <- c(trip_EM_par_step$sd1[i],
             (trip_EM_par_step$sd2[i]-trip_EM_par_step$sd2[i]/4),
             (trip_EM_par_step$sd3[i]+2))

  stepPar0 <- c(mu0,sigma0)
  angleMean0 <- c(pi,pi/2,0) # angle mean
  kappa0 <- c(0.1,0.2,0.8) # angle concentration
  anglePar0 <- c(angleMean0,kappa0)
  stepDist <- "gamma"
  angleDist <- "wrpcauchy"

  m <- fitHMM(data=out.list5hmm[[i]], nbStates=3, stepPar0=stepPar0,
               verbose=0, stepDist=stepDist, anglePar0=anglePar0, angleDist=angleDist)
  ks <- as.data.frame(unlist(m$mle))
  vit <- viterbi(m)

if (sum(ks) != 0 ) {
  write.table(ks, paste0(trip_EM_par_step$ID[i],".txt"))
  write.table(vit, paste0('viterbi_angle',trip_EM_par_step$ID[i],".txt"))
  print(paste("i=",i))
  plot(m, compact=TRUE, ask=FALSE) # the compact=TRUE puts all vessels in one graph

  } else {

      print(paste("i=",i, "is 0. Not processed")) # print out progress
      next
    }
}

dev.off()
graphics.off()

list.filenames <- list.files(path = ".", full.names = FALSE, pattern="^viterbi_angle")

datalist <- map(list.filenames, function(x) read.table(x, header=T, sep=""))
names(datalist) <- list.filenames
trial <- do.call(rbind.data.frame, datalist)
prepdat$viterbi_speed_angle <- trial$x
prepdat$trip_HMM_speed_angle_beh <- ifelse(prepdat$viterbi_speed_angle==1,
                                           "hauling", "not_hauling")

names(prepdat)
prepdat <- prepdat %>% select(-c("viterbi_speed","viterbi_speed_angle"))

# check number of correctly assigned behaviours for each position

names(prepdat)
prepdat <- prepdat %>%
  mutate(behaviour_simple = ifelse(prepdat$behaviour=="hauling",
                                   "hauling", "not_hauling"))
```

```r
prepdat <- prepdat %>%
    mutate(match_overall_EM_beh = ifelse(
            prepdat$overall_EM_beh==prepdat$behaviour_simple, 1,0),
           match_trip_EM_beh = ifelse(
            prepdat$trip_EM_beh==prepdat$behaviour_simple,  1,0),
           match_trip_EMbc_beh = ifelse(
            prepdat$trip_EMbc_beh==prepdat$behaviour_simple, 1,0),
           match_trip_HMM_speed_beh = ifelse(
            prepdat$trip_HMM_speed_beh==prepdat$behaviour_simple, 1,0),
           match_trip_HMM_speed_angle_beh = ifelse(
            prepdat$trip_HMM_speed_angle_beh==prepdat$behaviour_simple, 1,0))

sum(prepdat$match_overall_EM_beh) #2000
sum(prepdat$match_trip_EM_beh) #2021
sum(prepdat$match_trip_EMbc_beh) #2039
sum(prepdat$match_trip_HMM_speed_beh) #2004
sum(prepdat$match_trip_HMM_speed_angle_beh) #2028
```
